# A field trial of a PCR-based *Mansonella ozzardi* diagnosis assay detects high-levels of submicroscopic *M. ozzardi* infections in both venous blood samples and FTA® card dried blood spots

**DOI:** 10.1186/s13071-015-0889-z

**Published:** 2015-05-20

**Authors:** Jansen Fernandes Medeiros, Tatiana Amaral Pires Almeida, Lucyane Bastos Tavares Silva, Jose Miguel Rubio, James Lee Crainey, Felipe Arley Costa Pessoa, Sergio Luiz Bessa Luz

**Affiliations:** Laboratory of Entomology, Fiocruz Rondônia, Porto Velho, RO Brazil; Research Programme on Infectious Disease Ecology in the Amazon (RP-IDEA), Leônidas & Maria Deane Institute – Fiocruz Amazônia, Manaus, AM Brazil; Health and Endemic Diseases in the Amazon, Multi Institutional Graduate Program, Federal University of Amazonas (UFAM), Manaus, AM Brazil; Malaria & Emerging Parasitic Diseases Laboratory, Parasitology Department, National Centre of Microbiology. Carlos III Health Institute, Madrid, Spain

**Keywords:** Mansonelliasis, *Mansonella ozzardi*, PCR-detection, Submicroscopic, FTA®cards

## Abstract

**Background:**

*Mansonella ozzardi* is a poorly understood human filarial parasite with a broad distribution throughout Latin America. Most of what is known about its parasitism has come from epidemiological studies that have estimated parasite incidence using light microscopy. Light microscopy can, however, miss lighter, submicroscopic, infections. In this study we have compared *M. ozzardi* incidence estimates made using light microscopy, with estimates made using PCR.

**Methods:**

214 DNA extracts made from Large Volume Venous Blood Samples (LVVBS) were taken from volunteers from two study sites in the Rio Solimões region: Codajás [n = 109] and Tefé [n = 105] and were subsequently assayed for *M. ozzardi* parasitism using a diagnostic PCR (Mo-dPCR). Peripheral finger-prick blood samples were taken from the same individuals and used for microscopic examination. Finger-prick blood, taken from individuals from Tefé, was also used for the creation of FTA®card dried blood spots (DBS) that were subsequently subjected to Mo-dPCR.

**Results:**

Overall *M. ozzardi* incidence estimates made with LVVBS PCRs were 1.8 times higher than those made using microscopy (44.9 % [96/214] compared with 24.3 % [52/214]) and 1.5 times higher than the PCR estimates made from FTA®card DBS (48/105 versus 31/105). PCR-based detection of FTA®card DBS proved 1.3 times more sensitive at diagnosing infections from peripheral blood samples than light microscopy did: detecting 24/105 compared with 31/105. PCR of LVVBS reported the fewest number of false negatives, detecting: 44 of 52 (84.6 %) individuals diagnosed by microscopy; 27 of 31 (87.1 %) of those diagnosed positive from DBSs and 17 out of 18 (94.4 %) of those diagnosed as positive by both alternative methodologies.

**Conclusions:**

In this study, Mo-dPCR of LVVBS was by far the most sensitive method of detecting *M. ozzardi* infections and detected submicroscopic infections. Mo-dPCR FTA®card DBS also provided a more sensitive test for *M. ozzardi* diagnosis than light microscopy based diagnosis did and thus in settings where only finger-prick assays can be carried-out, it may be a more reliable method of detection. Most existing *M. ozzardi* incidence estimates, which are often based on light microscope diagnosis, are likely to dramatically underestimate true *M. ozzardi* parasitism incidence levels.

## Background

*Mansonella ozzardi* is a New World human filarial parasite that is broadly distributed throughout Central and South America. Locally acquired *M. ozzardi* infections have been reported in a diverse range of communities throughout Latin America, spanning from Mexico (in the north) to Argentina (in the south) [1]. Parasite incidence surveys have shown population parasitism levels in excess of 15 % in multiple Caribbean islands, Argentina, Bolivia, and numerous geographically diffuse localities within the Brazilian Amazon [2–8].

Together with its African relatives (*M. perstans* and *M. streptocerca*), *M. ozzardi* is one of three aetiological agents that causes human mansonelliasis [9, 10]. While many symptoms have been attributed to the mansonelliasis condition, there is presently no universally agreed symptom-set used for its clinical diagnosis [1, 9, 10]. Despite the fact that some of these attributed pathologies are quite disabling, the perception that mansonelliasis is mostly benign, seems to be the most prevalently held view by international policy makers. Certainly, the condition is not presently the subject of any major international or even national control programmes and is not presently regarded by the World Health Organisation (WHO) as one of the world’s 17 most Neglected Tropical Diseases [11].

Whether or not this general perception of Mansonelliasis as benign is justified, there is undoubtedly a strong and growing case that *M. ozzardi* parasitism in the Amazon region is of more medical importance than it is elsewhere. As well as the continuing problems concerning the discrimination of *O. volvulus* and *M. ozzardi* parasites and also exposure to these parasites in the Amazonia onchocerciasis focus [12–14], there is also a growing body of evidence to suggest that *M. ozzardi* infections can themselves directly cause articular pain, headaches, and in the Brazilian Amazon even that they can cause ocular lesions [2, 15]. Interest in how *M. ozzardi* parasitism may be affecting other parasitic infections, such as malaria, is also growing [4, 16–18]. The number of epidemiological studies, designed to detect correlations between the clinical presentation of symptoms of Mansonella parasite infections and behavioural risk factors associated with them, is thus rising [2, 5–7]. Fundamental to the success of all such studies is accurate diagnosis of parasitism, which for *M. ozzardi*, is traditionally done using light microscopy.

Molecular studies of other insect-borne blood parasites, such as malaria, have shown that incidence estimates based on microscopy alone can dramatically underestimate the true levels of parasitism in a population [19–21]. Typically, in malaria parasitism incidence studies, light microscopy will predict incidence levels half those predicted when PCR and microscopy are used in combination [19–21]. Most experts attribute the differences between the incidence estimates largely to differences in sensitivities between the two techniques: with microscopy only detecting heavy infections and PCR detecting both heavy and lighter (submicroscopic) infections. One Malaria expert has, for example, estimated that a good microscopist has a parasite detection threshold of about 40–50 parasites per micro litre, whereas PCR can be expected to routinely detect parasite densities lower than one parasite per micro litre [22].

In the study presented here we have set out to assess if light-microscope based diagnosis of *M. ozzardi* is under-reporting true *M. ozzardi* incidence levels and to collect data that may help improve the specificity and sensitivity of *M. ozzardi* diagnosis in the future. To achieve our objectives, we have used PCR and light-microscopy to diagnose the infection-status of 214 individuals in a *M. ozzardi* endemic region of the Brazilian Amazon. In a 105-person sub-sample of these individuals we have PCR-tested both a frozen venous blood-sample and a finger-prick FTA®card dried blood-spot as a way of assessing the best practice for sample preservation. By comparing the diagnoses made by the variously trialled parasite-detection methodologies, our study has revealed a very high incidence of submicroscopic *M. ozzardi* infections in the Brazilian Amazon and has provided important base-line data from which new more effective *M. ozzardi* diagnostic techniques may be developed.

## Methods

### Study site selection

**Fig. 1:**
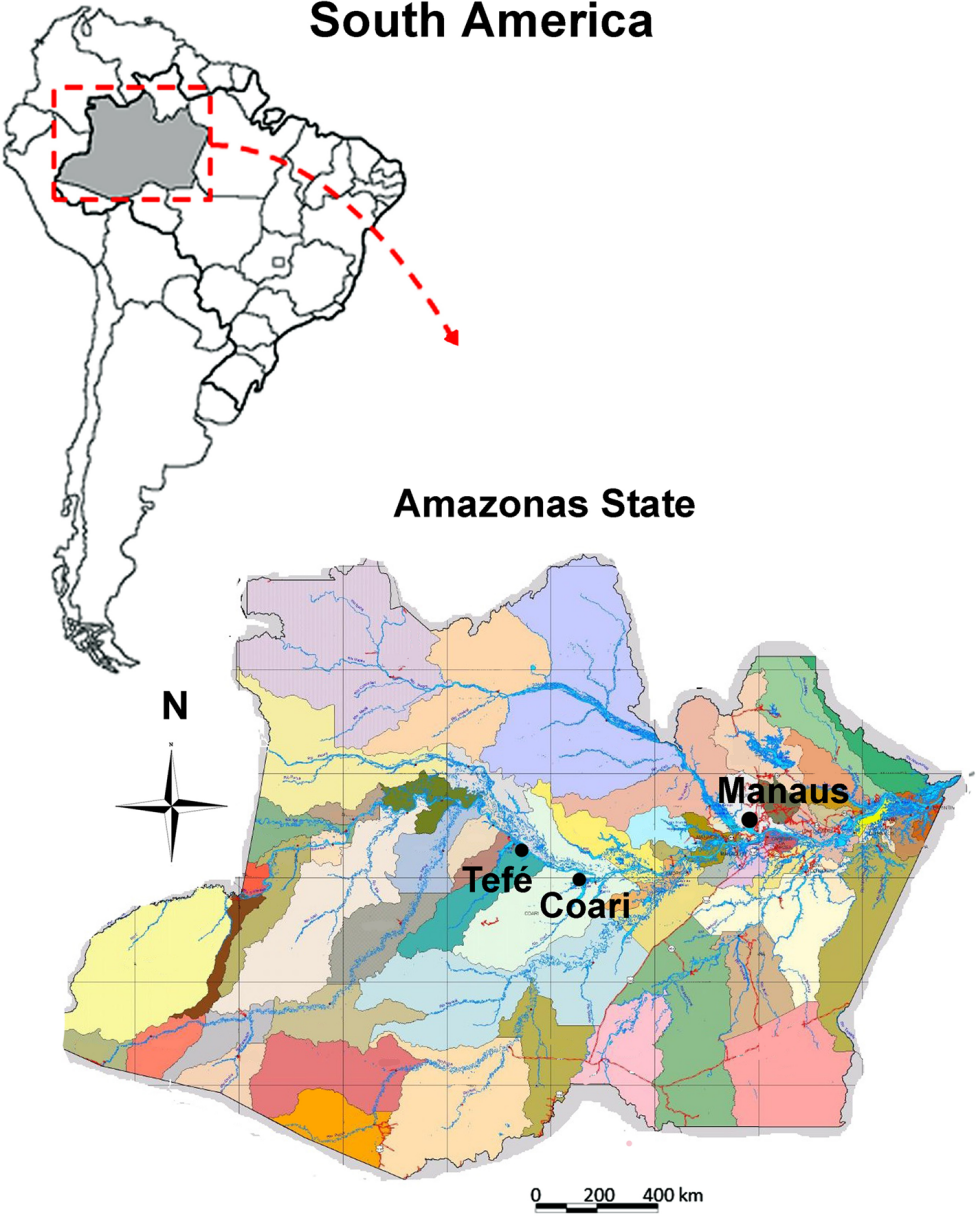
A map showing the location of the Tefé and Codajás localities used in this study. Amazonas state is shown inside of continental South America. These two study areas are shaded in grey and labelled appropriately; the Rio Solimões is shown in red

Very high levels of *M. ozzardi* parasitism have been recorded throughout the Brazilian Amazon region, but the Rio Solimões region e.g. Coari, Codajás, and Tefé region is one of only a few regions where high *M. ozzardi* incidence measurements and ocular pathologies have been recorded [7, 13]. In this study, blood samples were taken from two study sites around the Rio Solimões, referred to here as: (I) Tefé and (ii) Codajás. In each rural community, all residents were invited to participate voluntarily in the study. Volunteers who participated at the Tefé study-site arm of the study were from settlements close to Tefé. Participants from the Codajás study site were from settlements flanking the Rio Solimões between the towns of Codajás and Coari (see Fig. 1).

### Blood sampling

Blood samples were taken in one of two ways: peripheral blood was taken by digital finger puncture; venous blood samples were collected using a BD™ Vacuntainer system. Blood samples were collected from volunteers from the two study areas, following the procedure set out by the Research Ethical Committee of the Tropical Medicine Foundation Dr Heitor Vieira Dourado (in Manaus protocol 1504/10). Venous blood samples (of approximately 10 ml) were initially stored with EDTA at temperatures between 4 °C and 8 °C and then transferred to −20 °C before molecular processing. The thick blood smear preparations that were used for light microscopic parasite diagnosis were performed with peripheral blood samples shortly after the samples were taken. Dried Blood Spots (DBS) were also prepared from peripheral blood samples, but were only taken from the 105 volunteers from the Tefé study site.

### Light-microscopic detection of *M. ozzardi* infections

Light microscopy *M. ozzardi* diagnosis was performed on fresh peripheral blood samples obtained by finger punctures. Thick blood smears were made as previously described by Medeiros et al. [7].

### PCR detection of *M. ozzardi* infections

*Mansonella ozzardi* diagnostic PCRs (Mo-dPCRs) were carried-out following the amplification and band-scoring methodology detailed in Tang et al. [23]. As before, 5 μl of template DNA was used in an initial 50 μl PCR reaction mix; however, in this study the assayed template DNA was prepared slightly differently. For the large volume venous blood samples (LVVBS), the assayed DNA was derived from a 200 μl QIAGEN extract prepared using a QIAGEN blood and tissue extraction kit and protocol and an initial 100 μl of venous blood sample. For the Dried blood spots Mo-dPCRs, the initial template DNA was derived from a 150 μl Chelex®100-extract obtained from 20 (1.2 mm diameter) discs taken from a single peripheral blood drop (~20 μl) preserved on FTA®cards. Each volunteer’s 20 FTA®card discs samples were processed simultaneously using the manufacturer’s FTA®card neutralising solution and protocol. Processed discs were then incubated with 150 μl of 5 % Chelex®100 solution at 56 °C for 30 minutes and then at 100 °C for 10 minutes. A sample of 20 *M. ozzardi* positive samples were confirmed as such by forward and reverse Sanger sequencing using a methodology based on that described by Tang et al. [23].

All PCRs were performed alongside negative and positive controls. *Mansonella ozzardi* positive DNA preparations used in Tang et al. [23] were used in this study as positive controls. Negative controls were prepared with the template DNA being replaced by sterilised Millipore water and blood extract from volunteers whose blood had already repeatedly tested negative on previous microscope and PCR testing. *Mansonella ozzardi* PCR negative DNA preparations that tested positive for *M. ozzardi* in one or more other assays, were tested for the existence of amplifiable human DNA with routinely-used house-keeping gene primers, which specifically amplify the human β-actin gene [24]. FTA®card discs were cut using a Harris® 1.2 mm micro-punch instrument, with discs cut from blank areas of FTA®cards between DBS samples. These blank discs were also processed with FTA®card neutralising fluid and Chelex®100-extraction and served as further negative controls. These negative controls tested for Harris® micro-punch carry-over sample contamination.

## Results

*Mansonella ozzardi* and only *M. ozzardi* parasitism was detected by both traditional light microscopy and PCR at both study sites. Sequences of PCR products used for parasite diagnosis confirmed the existence of the parasite at both sites and found no evidence to support the existence of other filariae. All DNA preparations that tested positive in their first PCR assay also tested positive in their repeated PCR assay and no evidence of Harris® micro-punch carry-over was detected from the carry-over negative controls.

Table 1 shows a suite of sensitivity estimates calculated for each of the tested *M. ozzardi* diagnostic assays. A total of five sensitivity calculations have been made for each of the tested assays. Each of the five different sensitivity calculations assume a different method of defining whether a sample is truly positive or not (as indicated in Table 1). Regardless of which methodology is used as a reference “gold standard” of *M. ozzardi* infection, the PCR assay of LVVBS is calculated to be the most sensitive method of diagnosis. The lowest sensitivity estimate for this assay that we calculated was 83.1 % and the highest was 94.4 %. The 83.1 % sensitivity estimate for LVVBS-PCR diagnosis was calculated on the basis that an individual is indeed “truly positive” if they tested positive by any other method of diagnosis. All of the diagnostic tests assessed in the study using this definition of a “true positive” produce conservative sensitivity estimates (Table 1). This is because this method of estimating sensitivity is most likely to detect an assay’s false-negative reporting. It is, however, also the method most likely to underestimate an assay’s true sensitivity, as it is also the calculation most likely to over-estimate the number of “true positives” in a sample (because it neglects the possibility that other diagnostic assays could be generating false-positives).

**Table 1:** Sensitivity estimates for three *M. ozzardi* blood parasite detection assays

Parasitism reference standard	Venous blood ModPCR assay	Dried blood spot MoPCR assay	Light microscopy assay
Samples testing positive with the venous blood ModPCR assay		27/47 (57.4 %) [105]	21/47 (44.7 %) [105]
			44/96 (45.8 %) [214]
Samples testing positive with dried blood spot MoPCR assay	27/31 (87.1 %) [105]		18/31 (58.1 %) [105]
Samples testing positive with the light microscopy assay	21/24 (87.5 %) [105]	18/24 (75 %) [105]	
	44/52 (84.6 %) [214]		
Samples testing positives with any other assay	31/37 (83.8 %) [105]	28/50 (56 %) [105]	24/52 (46.2 %) [105]
	54/65 (83.1 %) [214]		47/101 (46.5 %) [214]
Sample testing positives with both other assays	17/18 (94.4 %) [105]	17/21 (81 %) [105]	17/27 (63 %) [105]
Samples testing positive with all three assays	47/53 (88.7 %) [105]	31/53 (58.5 %) [105]	24/53 (45.3 %) [105]
	96/107 (89.7 %) [214]		52/107 (48.6 %) [214]
Assay sensitivity-estimate ranges	83.1 %–94.4 %	56 %–81 %	44.7 %–63 %

Table 1 shows sensitivity estimates for three *M. ozzardi* blood parasite detection assays. Sensitivity calculations have been made using six different blood parasitism reference standards (as indicated). The number of blood samples which had test results included for the sensitivity estimates is given in square brackets. Estimates are divided into: (i) those based solely on data obtained from individuals from Tefé (for which data was obtained using all three assays) and for which 105 blood samples were assessed and (ii) the total datasets, which combines the data from Codajás with the Tefé data (for which there was a total 214 blood samples assessed)

Although some of the light microscopy sensitivity estimates that we calculated are higher than some of the DBS ModPCR estimates, the sensitivity estimates that we made for DBS were always higher than the directly comparable estimates for light microscopy made using the same reference standard for what is a “truly” infected individual (Table 1). Our study data thus strongly support the notion that PCR based diagnosis (performed on DBS or LVVBS) is a lot more sensitive than microscopy based diagnosis of thick blood smears.

It is important to note, however, that all three assays used to diagnose *M. ozzardi* infections provided strong evidence of reporting false negatives (Table 1). Even if the most stringent conditions of what is a “truly positive” individual is assumed and thus that only those testing positive with two or more assays are regarded as “truly positive”, all three methods of diagnosis can be seen to have reported false negatives (Table 1). Although, our results suggest that LVVBS is substantially more sensitive than thick blood smear methods of light microscopy diagnosis, in its present form at least, it should not properly be regarded as a true gold standard of diagnosis. As we also have no reason to suspect that any of our diagnosed positives are false positives, these calculations only serve to illustrate the limitations of using thick blood smears and light microscopy to estimate *M. ozzardi* parasitism population incidence levels.

In terms of understanding of *M. ozzardi* epidemiology in the Rio Solimões region, overall incidence rates almost doubled with the inclusion of PCR-based diagnosed individuals from 52 out of 214 volunteers (24.3 %), (based on light microscopy alone) to 100 out of the same 214 (46.7 %) volunteers when both light microscopy and PCR diagnoses were combined, showing a 1.92 fold increase in incidence estimates (Table 2). Underlying this was a very large number of individuals that tested positive only by PCR-based methods of diagnosis. Such sub-patent or submicroscopic infections accounted for 48 of the 214 volunteers (22.4 %) of all those diagnosed as *M. ozzardi* infected (Table 2). At both study sites, and using both LVVBS, and DBS, our analysis detected more parasite positive samples using the PCR based diagnosis method than by using the light microscopy based method (Table 2). Overall parasitism incidence estimates for the Rio Solimões region can be increased by 1.85 times if LVVBS ModPCR is used as the method of diagnosis instead of light microscopy: LVVBS ModPCR estimated incidence levels of 44.9 % in contrast to the 24.3 % incidence estimates made using light microscopy from the same volunteers (Table 2). ModPCR also managed to detect 1.29 times more parasitized individuals than light microscopy did for the same set of 105 volunteers from Tefé (Table 2), suggesting it is a more sensitive technique than light microscopy based diagnosis used in isolation.

**Table 2:** *M. ozzardi* prevalence estimates for the river Rio Solimões region

	Codajás	Tefé	Rio Solimões combined data
Venous blood ModPCR prevalence estimates	49/109 (45 %)	47/105 (44.8 %)	96/214 (44.9 %)
Dried blood spot ModPCR prevalence estimates		31/105 (29.5 %)	
Light microscopy prevalence estimates	25.7 % (28/109)	22.9 % (24/105)	24.3 % (52/214)
Sub-microscopic infection prevalence estimates	VB-ModPCR: 20.2 % (22/109)	VB-ModPCR: 24.8 %(26/105)	VB-ModPCR: 22.4 % (48/214)
		DBS-ModPCR: 11.4 %(12/105)	DBS or VB ModPCR: 23.8 % (51/214)
		DBS or VB ModPCR: 27.6 %(29/105)	
Combined data prevalence estimates	VB-ModPCR & LM: 45.9 % (50/109)	VB-ModPCR: 47.6 % (50/105)	VB-ModPCR: 46.7 % (100/214)
		DBS-ModPCR: 34.3 % (36/105)	DBS or VB ModPCR: 48.6 % (104/214)
		DBS or VB ModPCR: 51.4 % (54/105)	
Light microscopy false negatives	VB-ModPCR & LM: ≥27.2 % (≥22/81)	VB-ModPCR: ≥32.1 % (≥26/81)	VB-ModPCR: 2.08
		DBS-ModPCR: ≥14.8 % (≥12/81)	DBS-ModPCR: 1.5
		DBS or VB ModPCR:≥35.8 % (≥29/81)	DBS or VB ModPCR: 2.25
Light microscopy prevalence underestimating	VB-ModPCR & LM: 1.79 ×	VB-ModPCR: 2.08 ×	VB-ModPCR:1.92 ×
		DBS-ModPCR: 1.5 ×	DBS or VB ModPCR: 2 ×
		DBS or VB ModPCR: 2.25 ×	

Table 2 shows *M. ozzardi* summary prevalence estimates for the Rio Solimões as a whole and divided by the two study areas. Estimates are also shown divided into infections that were detected (patent) in light microscopy and infections that were not detected by light microscopy, but were detected by PCR: sub-microscopic infections. Additionally, the table shows a breakdown of sub-microscopic parasite detection and prevalence estimates made by different assaying methods. Corresponding calculations based on each assay's submicroscopic parasite detection have also been used to estimate the minimum number of light microscopy (LM) false negatives and prevalence underestimating for *M. ozzardi* parasite infections. All calculations neglect the possibility of false positives. Dried blood spot is abbreviated to DBS and venous blood to VB

## Discussion

In this study we have shown that *M. ozzardi* parasitism incidence estimates in the Rio Solimões region, made using light microscopy-based diagnosis in isolation, are about half those made using the method in combination with ModPCR assays. A similar under-reporting of sub-patent or submicroscopic blood parasite infections has been described for malaria infection diagnosis in a diverse range malaria endemic settings [16–18]. It seems likely that the light microscope *M. ozzardi* parasitism incidence underestimations made in this study are typical of light microscopy based *M. ozzardi* parasitism surveys. As most existing *M. ozzardi* incidence estimates (and indeed other Mansonella parasitism incidence estimates) are based on light microscope diagnosis, most *M. ozzardi* incidence estimates are therefore likely to be gross underestimates of true levels of *M. ozzardi* parasitism incidence levels in the Amazon region and in other areas beyond [2–8].

Mansonelliasis parasitism caused by *M. perstans* in Africa has been calculated to be affecting 114 million people [25]. These estimates have been made on the basis of ~20 % incidence levels (a value typical of *M. perstans* light microscopy incidence surveys) [25]. To our knowledge, no such global estimates of *M. ozzardi* parasitism have been recently calculated; however, incidence measurements of about 20 % are typical of traditional light-microscopy surveys and are in-line with what we have observed. Hopefully, our results will ensure that if such *M. ozzardi* global estimates are made in future they will take into account submicroscopic infections and by doing so could potentially double estimates made solely from light microscopy incidence surveys alone. In a recent Global Burden of Disease (GBD) survey, which uses disease pathology, and incidence to estimate the public health impact of disease, Mansonelliasis infections were not listed among the 291 catalogued diseases that had their global impact assessed [26]. This is probably because Mansonelliasis is often regarded as completely benign and/or because the pathologies that have been attributed to Mansonella infections are so poorly defined, making meaningful calculations of the burden of Mansonelliasis presently infeasible. Our results suggest, however, that if Mansonelliasis does have a clinical pathology and is included in a future global burden study, it could be seen to have a much greater burden than would have been projected from traditional blood-smear based light-microscopy diagnosis alone.

Most of the risk factors and clinical symptoms that have previously been attributed to *M. ozzardi* infections have come from epidemiological studies that have reported co-incidences of infections and volunteers’ sex, age and/or reported symptoms, and/or demographics [2, 5, 7, 15]. Similar surveys have, however, often reported conflicting results and as things stand the only undisputed medical significance of *M. ozzardi* parasites and their infections is that they can be confused with *O. volvulus* parasites and their infections and that this can interfere with onchocerciasis research and control [1, 12, 23]. Our results show that many of the individuals diagnosed as negative using light microscopy are in fact positive, but positive with sub-patent submicroscopic infections. If Mansonelliasis does have a clinically important pathology, our results could help to explain why *M. ozzardi* parasitism has remained so ill-defined. For example, it is well understood (from extensive light microscopy diagnosed infection surveys) that the levels of patent *M. ozzardi* infections can vary dramatically across space and time [2, 5–7]. Thus it is possible that submicroscopic infections vary similarly (but asynchronously with respect to patent infections) and that the incongruence between similar *M. ozzardi* epidemiological studies, may be explained by simple inconsistent levels of misdiagnosis in epidemiological surveying. *Mansonella ozzardi* epidemiological surveys using both light microscopy and ModPCRs in combination thus offer the possibility to re-evaluate the clinical importance of *M. ozzardi*. It is also clear from this work that combining light microscopy and ModPCR provides the potential to make a convenient subdivision between light levels of parasitism (submicroscopic infections) and heavy levels of parasitism (microscopically patent) as is already carried out in Malaria research [19–22]. This could potentially help make the clinical symptoms of the infections as well as the risk factors for contracting the parasites become more salient.

From a technical perspective, our study showed that the Tang et al. [23] ModPCR assay used in this study is more sensitive for parasite detection than light microscopy alone― even when only small peripheral finger-prick blood samples are available. DNA preparations made from 100 μl LVVBS were shown in this study to be substantially more effective at detecting parasite infections than the alternative tested methods (Table 1). There is, however, little doubt that this approach was also missing true positives, that is to say individuals infected with *M. ozzardi*. The LVVBS ModPCR assay should not therefore be regarded as a true gold standard, even though our data suggests it is the best reported assay for detecting *M. ozzardi* parasitism.

Blood samples are notorious for containing PCR inhibitors and so it is unlikely that ModPCR tested in this study can be expected to identify all truly positive individuals [27]. However, in the case of this study PCR inhibitors are unlikely to account for our “false negative” results as all of our false negative results had human DNA successfully amplified from them, showing both that the DNA extraction procedure in the sample processing had worked and that PCR inhibitors were absent or in limited quantities. Our results thus suggest that there is scope for both of our ModPCRs-based assays to be improved.

In previous parasite assaying studies, increasing the total amount of starting whole blood used to prepare a DNA extract for PCR detection was shown to increase the assay’s sensitivity [27]. Consistent with this, the LVVBS assay used in this study contained more starting DNA than the DBS PCRs and also showed higher sensitivity. Although the differences in our study findings might also be explained by differences in pre-processing methodologies, both the preservation methodologies used in this study are routinely used methods for preserving DNA for PCR. It thus seems likely that the sensitivity of both assays could be improved by increasing the volume of whole blood used in the assay extraction procedures. In other studies of parasitism diagnosis techniques, the sensitivity of the assays has been shown to be increased without the need to sample more initial staring material by, for example, the lysis of parasites prior to their application to FTA®cards [27]. Because most of the proven methods of liberating DNA from microfilariae (which have tough cuticles) involve processes that are not easily performed in a field setting (like, for example, laser-dissection, and liquid-nitrogen freeze-thawing), the most practical ways of improving the sensitivity of ModPCR-based *M. ozzardi* diagnosis techniques, are thus likely to be achieved by assaying larger volumes of whole blood [28].

While other ModPCRs could be trialled to improve *M. ozzardi* diagnosis, none of the existing published methods have yet been validated to the extent of the Tang et al. method [23]. Some of the published *M. ozzardi* PCR assays, for example, still need to be tested on field material, while others still need to show the PCR products they amplify are indeed of *M. ozzardi* origin [29, 30]. As there is presently no reason to be believe that the Tang et al. [21] ModPCR used in this study is reporting false-positives or false-negatives (when *M. ozzardi* DNA is available to amplify), it appears to us that, at this stage at least, *M. ozzardi* diagnostics will improve most rapidly if ModPCR-based diagnosis continues to focus on its use.

## Conclusions

In this study, the ModPCR performed on LVVBS was by far the most sensitive method of detecting *M. ozzardi* infections and detected high-levels of submicroscopic infections. ModPCR detection of *M. ozzardi* parasites preserved within FTA®card DBS also provided a more sensitive test for *M. ozzardi* diagnosis than light microscopy, in situations where only finger-prick assays can be carried-out, it maybe be a more reliable method of detection. Most existing *M. ozzardi* incidence estimates, which are often based on light microscope diagnosis, are likely to be dramatically underestimating true *M. ozzardi* parasitism incidence levels. Both types of ModPCR assays tested in this study could potentially be improved by increasing the amount of whole blood starting material. Future ModPCR-based epidemiological studies could potentially give a more clear picture of true global *M. ozzardi* incidence levels and shed more light on the clinical symptoms of Mansonelliasis infections and also the risk factors associated with them.
